# Identification and characterization of the Chinese giant salamander (*Andrias davidianus*) miRNAs by deep sequencing and predication of their targets

**DOI:** 10.1007/s13205-017-0817-3

**Published:** 2017-07-10

**Authors:** Yong Huang, You Bing Yang, Xiao Chan Gao, Hong Tao Ren, Xi Hong Sun

**Affiliations:** College of Animal Science and Technology, Henan University of Science and Technology, Luoyang, 263 Kaiyuan Avenue, Luoyang, 471023 China

**Keywords:** Deep sequencing, *Andrias davidianus*, miRNAs, Targets

## Abstract

MicroRNAs (miRNAs) are non-coding small RNA of approximately 22 nt in length. They are considered as key regulators for gene expression at the post-transcriptional level and play an important role in the regulation of many fundamental biological processes. *Andrias davidianus*, as one of the largest amphibian on earth, may represent the transitional type of animal from aquatic to terrestrial life, but so far, no miRNA has been identified in the species. In this study, Illumina deep sequencing was used for high-throughput analysis of miRNAs in a pooled small RNA library isolated from eight tissues sample of *A. davidianus* including the spleen, liver, muscle, kidney, skin, testis, gut, and heart. In total, 6,213,146 reads were obtained, 5,962,175 of which were related to 143 unique miRNAs, including 140 conserved and three novel *A. davidianus*-specific miRNAs. Among them, seven conserved miRNAs and one novel miRNA were selected to validate their expression pattern by stem-loop qRT-PCR. Moreover, 4700 potential target genes were predicted for 143 *A. davidianus* miRNAs; GO enrichment analysis and KEGG pathway showed that most of the targets are involved in diverse biological processes including ubiquitin-mediated proteolysis, FOXO signaling pathway, tight junction, and spliceosome. This study provides the first large-scale identification and characterization of *A. davidianus* miRNAs, and predicted their potential target genes; it will lay a valuable foundation for future understanding the role of these miRNAs on regulating diverse biological processes.

**Electronic supplementary material:**

The online version of this article (doi:10.1007/s13205-017-0817-3) contains supplementary material, which is available to authorized users.

## Introduction

MicroRNAs (miRNAs) are a class of genome-encoded small RNAs with approximately 22 nt in length, that are abundant in nearly all metazoans, plant, fungi, protists, and even in viruses (Lin et al. 2009; Li et al. 2010b; Sun et al. 2012; Mu et al. 2015; Chen et al. 2016; Huang and Evans 2016). Biogenesis of miRNAs, in general, starts from the transcription of pri-miRNAs by RNA polymerase II in the nucleus, which are cleaved by Drosha to produce 60–80 nt-long stem-loop pre-miRNAs (Lee et al. 2004). Subsequently, pre-miRNAs are transported to the cytoplasm by exportin-5 protein and cleaved by another RNase enzyme (Dicer) to generate ~22 nucleotide double-stranded mature miRNAs (Bartel 2004). One of the two strands of the miRNA will be assembled with the RNA-induced silencing complex. These mature miRNAs can regulate the expression of target genes by binding to the coding regions or untranslated regions (Siomi and Siomi 2010). It has been estimated that they may target up to 30% of protein-coding genes in animals. Each miRNA may have multiple gene targets, and each gene target may also be regulated by more than one miRNA (Wen et al. 2012). Growing evidences indicate that miRNAs act as key regulators of genes which involved in the regulation of multiple biological processes such as tissue development, cellular proliferation and differentiation, immune response, gametogenesis, metastasis, signal transduction, and oncogenesis (Hobert 2008; Huang et al. 2011; Rateitschak et al. 2016; Sachdeva et al. 2016).

The Chinese giant salamander (*A. davidianus*) is the largest extant amphibian in the world (Murphy et al. 2000; Zhang et al. 2016). Now, it is classified as an endangered species by the International Union for Conservation of Nature and Nature Resources, and is the class II state major protection species in China. In the evolution history of vertebrates, the Chinese giant salamander occupies a seat at the phylogenetic and species evolution process which is representing a transitional form that links the aquatic animals to terrestrial organisms. Therefore, this species has an important scientific value. Over the past a few years, thousands of miRNAs had been identified through the application of direct clone, computational prediction, and deep sequencing technologies, and more than 28,645 miRNAs were annotated in miRBase database release 21.0 in June 2014 (http://www.mirbase.org/). However, little is known about the miRNA of *A. davidianus*. Currently, deep sequencing has become a powerful approach for high-throughput gene identification on a genome-wide scale in non-model organisms; it has an important advantage and can detect almost all small RNA such as known and novel miRNAs even with extremely low abundance (Hallman et al. 2013). In this study, we performed the first deep sequencing study of the miRNA population in *A. davidianus*, and identified 143 miRNAs, and predicted their gene targets; this information will be helpful for understanding of miRNA populations in *A. davidianus*, and lay a solid foundation for further functional studies of these miRNAs.

## Materials and methods

### Ethics statement and sample collection

The second generation of the farmed *A. davidianus* (4 years old) was obtained from Luoyang Huani Bio-Tech Co., Ltd (Luoyang, China). This study has also been reviewed and approved by the Ethics Committee of Henan University of Science and Technology according to the Regulations for the Administration of Affairs Concerning Experimental Animals (Ministry of Science and Technology, China; revised in June 2004). Subsequently, *A. davidianus* were anesthetized and sacrificed by decapitation. The spleen, liver, muscle, kidney, skin, testis, gut, and heart were sampled and stored in liquid nitrogen for further use.

### Constructing and sequencing of small RNA libraries

Total RNA was extracted from the mixed organs using TRIzol reagent (Invitrogen, USA) and then was treated with RNase-free DNase I (Takara, China) to remove any contaminating genomic DNA according to the manufacturer’s protocol. The quantity and purity of total RNA was measured by NanoDrop ND-1000 spectrophotometer (Nano Drop, USA) at 260/280 nm (ratio = 2.0). The integrity of total RNA was confirmed by Bioanalyzer 2100 and RNA 6000Nano LabChip Kit (Agilent, USA) with RIN number >7.0. Briefly, small RNAs of 18–35 nt in length were first isolated from the total RNA by size fractionation. Then, these small RNAs were ligated with 5′-RNA and 3′-RNA adapters, and subsequently, reverse transcription PCR was used to create cDNAs. The amplified cDNAs were purified and sequenced with Illumina HiSeq 2500 platform.

### In silico analysis of small RNA sequencing data

After deep sequencing, raw data were processed through Novogene Company’s Perl and Python scripts. In this step, clean data were obtained by removing the contaminating reads, sequences containing adapters, without insert tags and reads containing poly A or T or G or C. Sequences from 18 to 35 nt in length were selected for further analysis. Then, the retained reads were searched against the NCBI, Rfam, and Repbase database to remove known classes of RNAs (mRNA, rRNA, tRNA, snRNA, snoRNA, and repeats), so that every unique small RNAs mapped to only one annotation. Furthermore, small RNA reads from the exons of protein-coding genes were also excluded to avoid mRNAs contamination. The sequencing reads survived from the above strict filter rules were deemed to high-quality reads. Since there is no published genome information of *A. davidianus*, the high-quality reads were mapped to *Xenopus tropicalis* genome sequence using the Bowtie software (Langmead et al. 2009). The mappable small RNA tags were aligned to the miRNA precursor in the miRNA database (miRBase. 21.0; released in June, 2014) to obtain the known miRNA count. Finally, novel miRNAs were predicted by exploring the secondary structure, the Dicer cleavage site, and the minimum free energy of the former unannotated small RNA tags which could be mapped to the reference sequence by available software miRDeep2 (Friedlander et al. 2012). The generated raw reads have been deposited in NCBI’s SRA database under accession numbers PRJNA374625.

### Validation of miRNAs by stem-loop qRT-PCR

Total RNA was extracted using RNAiso Plus reagent (Takara, China) according to the manufacturer’s protocol. RNA quality was estimated with the nanophotometer (Implen, USA) and the 1% agarose gel electrophoresis. Stem-loop qRT-PCR was adopted to validate and measure miRNA expression. 1 μg of the total RNA and specific RT primer was reverse transcribed used forto stem-loop reverse transcription. Primers for stem-loop RT-PCR were designed according to the descriptions in prior studies (Varkonyi-Gasic and Hellens 2011), and all the primers are listed in Table S1. Specified qPCR was then performed on an ABI Step One Real-Time PCR System (Applied Biosystems, USA) using SYBR Premix Ex Taq (Takara, China). PCR conditions were as follows: heated at 95 °C for 15 min, followed by 40 cycles of 95 °C for 5 s, 58 °C for 10 s, and 72 °C for 10 s. All reactions were repeated three times. The 2^−ΔΔCT^ method for relative quantification of gene expression was used to determine the level of miRNA expression and 18SrRNA was used for normalizing the data.

### Prediction and functional annotation of the target genes of miRNAs

The position of 2–8 nt in a mature miRNA is called the seed region which is highly conserved, and this seed region most often binds to a target site in the target mRNA by perfect complementarity. The conserved and novel miRNAs of *A. davidianus* were used as query sequences to blast against sequences of *X. tropicalis* mRNA, and to predict target genes using target genes prediction software miRanda (http://www.microrna.org/microrna/home.do). The nucleotides 2–8 from the 5′end of miRNA will be retain. The energy threshold was set at ≤−20 kcal/mol and other thresholds used their default values. The predicted target genes were used for GO enrichment analysis (Young et al. 2010) and KEGG enrichment analysis (Kanehisa et al. 2008). KOBAS software (Mao et al. 2005) was used to test the statistical enrichment of the target gene candidates in KEGG pathways.

## Results and discussion

### Summary of small RNA library data set by deep sequencing

**Fig. 1:**
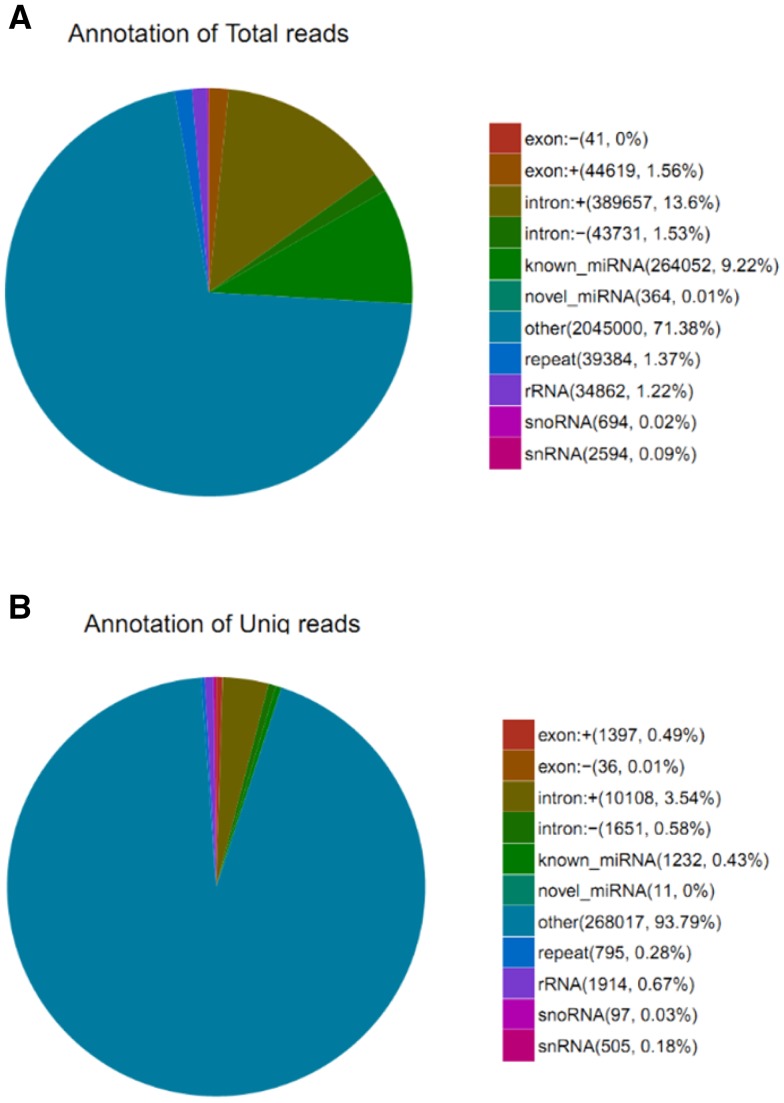
Distribution of small RNA among different categories in *A. davidianus*by deep sequencing. **a** Total reads; **b** unique reads

Deep sequencing is an ideal method for unbiased and unselected identification of miRNAs, and other ncRNAs in diverse species, especially in those that are less extensively studied. It does not require a priori knowledge of the sequence of the RNA species to be detected, but provides exact and quantitative sequence information. To identify miRNAs of *A. davidianus*, a small RNA library was constructed. As a result, a total of 6,213,146 raw reads were obtained. After removing contaminating sequences, 5,962,175 clean reads were generated (Table S2). For further study, 18–35 nt small RNA sequences were selected. The lengths of the most of the small RNAs were 21–24 nt (Fig. S1), and the most abundant size class in the small RNA sequence distribution was 11.38% (22 nt), followed by 7.79% (21 nt), 6.15% (23 nt), and 6.67% (24 nt), which are typical of small RNA Dicer-processed products and are consistent with the known 18–25 nt range for miRNAs. The length variations have been mainly attributed to enzymatic modification such as RNA editing, 3′-editing, and exonuclease activities (Li et al. 2010a). Because the genome sequence of *A. davidianus* is not available, so the *X. tropicalis* genome was used as a reference for the analysis, and it was downloaded from the NCBI database (http://www.ncbi.nlm.nih.gov/genome/?term=Xenopus). The remaining high-quality sequences were mapped to the reference genome using the SOAP software (Li et al. 2008). A total of 2,864,998 reads in *A. davidianus* were perfectly mapped to the reference genome; 285,763 reads of *A. davidianus* were mapped to the genome with no mismatch (Table S3 and Table S4). These small RNAs were then classified into several different categories according to their annotations. The sequences of rRNA, tRNA, snRNA, and snoRNA were separated from the miRNAs and discarded, which were identified using a basic local alignment search tool against the known non-coding RNAs deposited in the Rfam database and NCBI databases. We also removed repeat-associated RNA, which mediate the silencing of genomic repeats and transposons. Subsequently, the small RNA tags were also aligned on the exons and introns of mRNA to find the degraded fragment of mRNA in the small RNA tags. The distribution of small RNAs among different categories is shown in Fig. 1. Finally, the rest of small RNAs were used for the identification of conserved miRNAs and the prediction of novel miRNAs.

### Identification and characterization of the conserved and novel miRNAs

The conservation of miRNA sequences might indicate that their functions are conserved across different species. To identify conserved miRNAs in *A. davidianus*, small RNA sequences identified from deep sequencing were searched against miRBase 21.0 with Blastn program. A total of 140 known miRNAs belonging to 76 families were identified in *A. davidianus* (Table S5). Most of the *A. davidianus* miRNAs in our study were also reported across various animal species, it suggests that they have conserved roles. We analyzed members of *A. davidianus* miRNA families and found significant difference among them. The members in different families were differed greatly (Table S6). Let-7 and miR-10 represent the largest families with 9 and 8 members, respectively. Of the remaining, 12 families have more than 3 members, 24 families have 2–3 members, and 38 families are represented by only a single member. Abundant miRNAs play essential and broad regulatory function in biological processes. In the current study, the most highly expressed miRNA in *A. davidianus* was miR-148a (33,180) followed by miR-451 (17,840), miR-1a (17,438), miR-499 (17,086), miR-217 (12,852), and miR-200a (11,646). The previous studies have indicated that miR-148a is involved in a variety of cancers in mammals. For example, Yuan et al. found that miR-148a was up-regulated in liver of chronic hepatitis B virus (HBV) infection persons. Anti-miR-148a suppressed cell proliferation, cell cycle progression, cell migration, anchorage independent growth in soft agar, and subcutaneous tumor formation in SCID mice (Yuan et al. 2012). Moreover, Guo et al. demonstrated that miR-148a promoted cell proliferation by targeting p27 in gastric cancer cells (Guo et al. 2011).

**Fig. 2:**
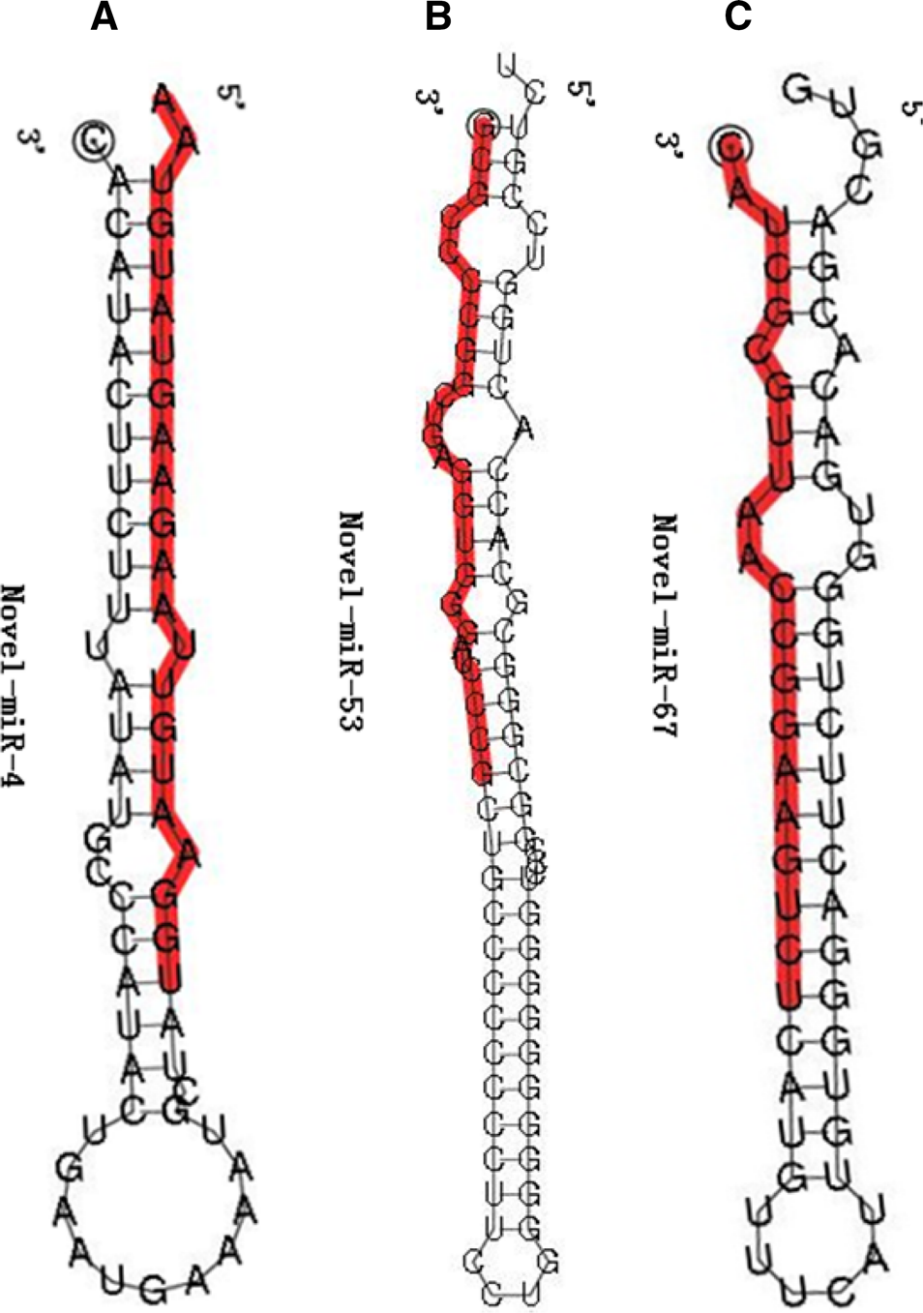
Predicted secondary structures of novel miRNAs in *A. davidianus.* Dominant forms of the mature miRNAs are indicated in *red*. **a** Novel-miR-4, **b** novel-miR-53, and **c** novel-miR-67

Deep sequencing can also be employed to detect novel miRNAs with low expression in small RNA transcriptomes (Fahlgren et al. 2009). To predict novel miRNAs in *A. davidianus*, all the mappable small RNAs were blasted to the *X. tropicalis* genome sequence in NCBI database with the known animal miRNAs in miRBase. The small RNAs that exactly map to the genome sequence but not map to the animal known miRNAs were classified as the candidate novel miRNAs. As a result, three novel miRNAs which belong to three miRNA families were identified in this study. The information about the numbers of reads and the sequence characteristics of three novel identified miRNAs using deep sequencing are summarized in Table 1. The formation of a stable hairpin structure was one of the essential features for the identification of new miRNAs (Fromm et al. 2015). The hairpin structures of the precursors of three novel identified miRNAs are shown in Fig. 2. The lengths of mature miRNAs were distributed in the range of 21–25 nt. The MFE of these predicted pre-miRNAs ranges from −49.1 to −21.0 kcal/mol. The MFEI ranges from 0.77 to 1.24 with an average of 0.98, which is consistent with the characteristics of miRNA. The results indicated that three novel identified miRNAs are also likely to be *A. davidianus*-specific miRNAs. Novel miRNAs in *A. davidianus* were predicted only three miRNA precursor sequence, but none of their mature miRNA counterparts (miRNA*) were predicted. This might due to the lack of related *A. davidianus* genomic information.

**Table 1:** *A. davidianus* novel miRNAs and their characteristics

miRNAs name	Gene source	Mature sequence	Size (nt)	Read count	Strand	L P (nt)	G + C (%)	MFE (kcal/mol)	MFEI
Novel-miR-4	GL173047.1	UGGAAUGUUAAGAAGUAUGUAA	22	334	Plus	62	27.3	−21.0	1.24
Novel-miR-53	GL173034.1	GCGCCCCGGCUGAGGUGGGAUCCCG	25	18	Plus	80	80.0	−49.1	0.77
Novel-miR-67	GL172912.1	CAUCGCGUUAACCGGAAGUCU	21	11	Minus	58	52.4	−28.2	0.93

### Nucleotide bias of identified miRNAs in *A. davidianus*

**Fig. 3:**
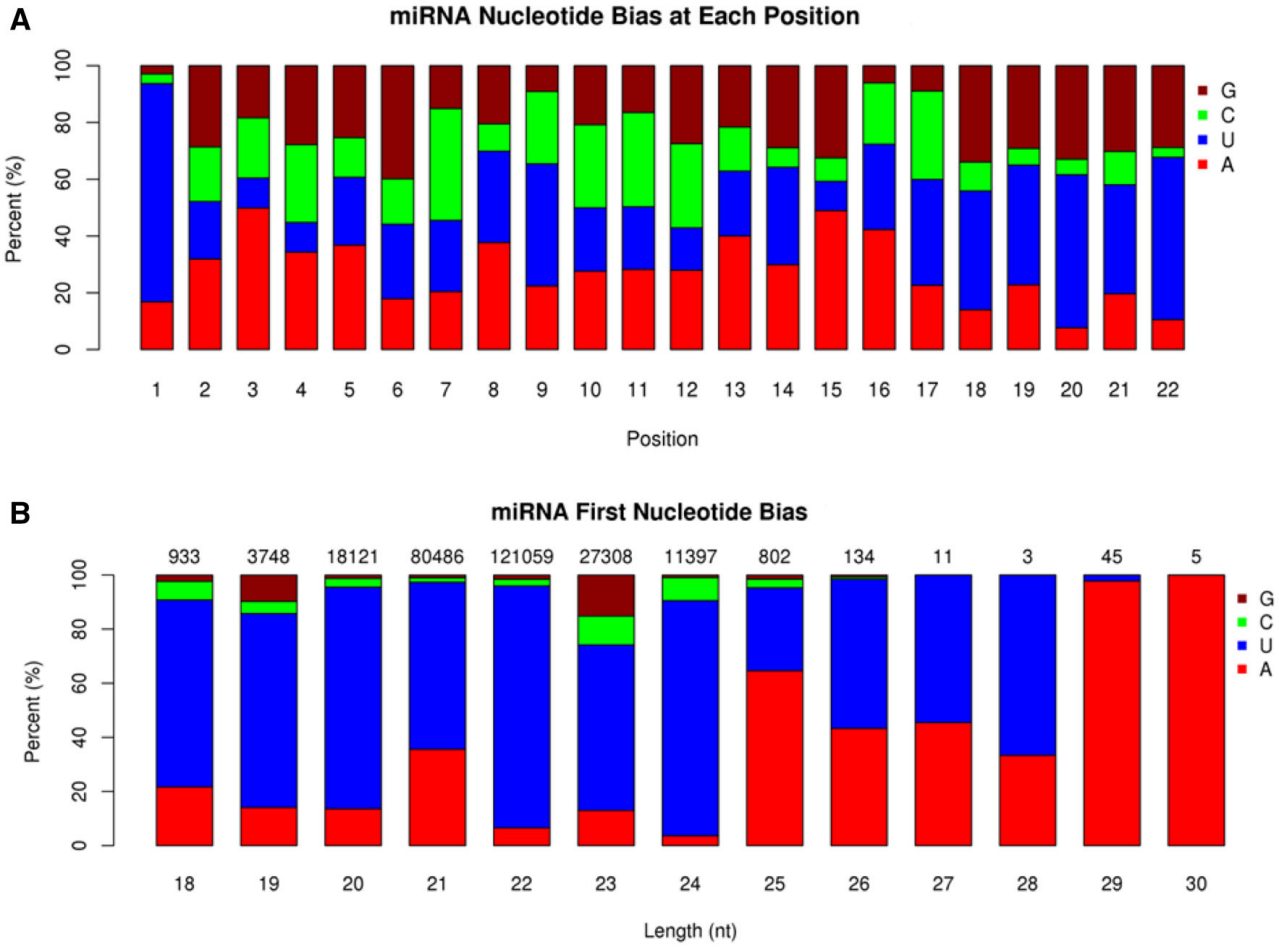
Base bias of miRNAs in *A. davidianus*. **a** Base bias on the first site of miRNAs with specific lengths; **b** base bias on the specific site of miRNAs

The percentage of the four nucleotides that appeared at each position of the identified miRNAs in *A. davidianus* was analyzed. U was the most frequent nucleotide in the first position (78.43%), followed by A (20.85%), while C or G was seldom used as the first nucleotide at only 3.45 and 2.86%, respectively (Fig. 3a; Table S7). Further analysis showed that U also had a high frequency at the 12th and 24th positions with percentages of 53.92 and 57.19%, but the lowest rates of 10.57, 10.45, and 10.30% at the 3rd, 4th, and 15th positions, respectively. The bias toward U at these positions may contribute to the regulatory mechanism of the molecule (Ge et al. 2013). Moreover, A + U dominated in the miRNA sequences and occupied a very high percentage at the start and the ends of reads, while a relatively high frequency of (G + C) appeared mostly at the middle of the reads. This phenomenon suggests that AU base paring may have effects on secondary structure or mRNA target recognition (Ji et al. 2012). In addition, we analyzed the first nucleotide bias in all identified miRNAs (Fig. 3b), and U was also the most abundant (69.13%) at the 5′end of all predicted miRNAs which is coordinated with the previous studies and an important characteristic of miRNAs in both plant and animals (Sun et al. 2013; Wang et al. 2014).

### Validation of the expression of the predicted miRNAs with stem-loop qRT-PCR

**Fig. 4:**
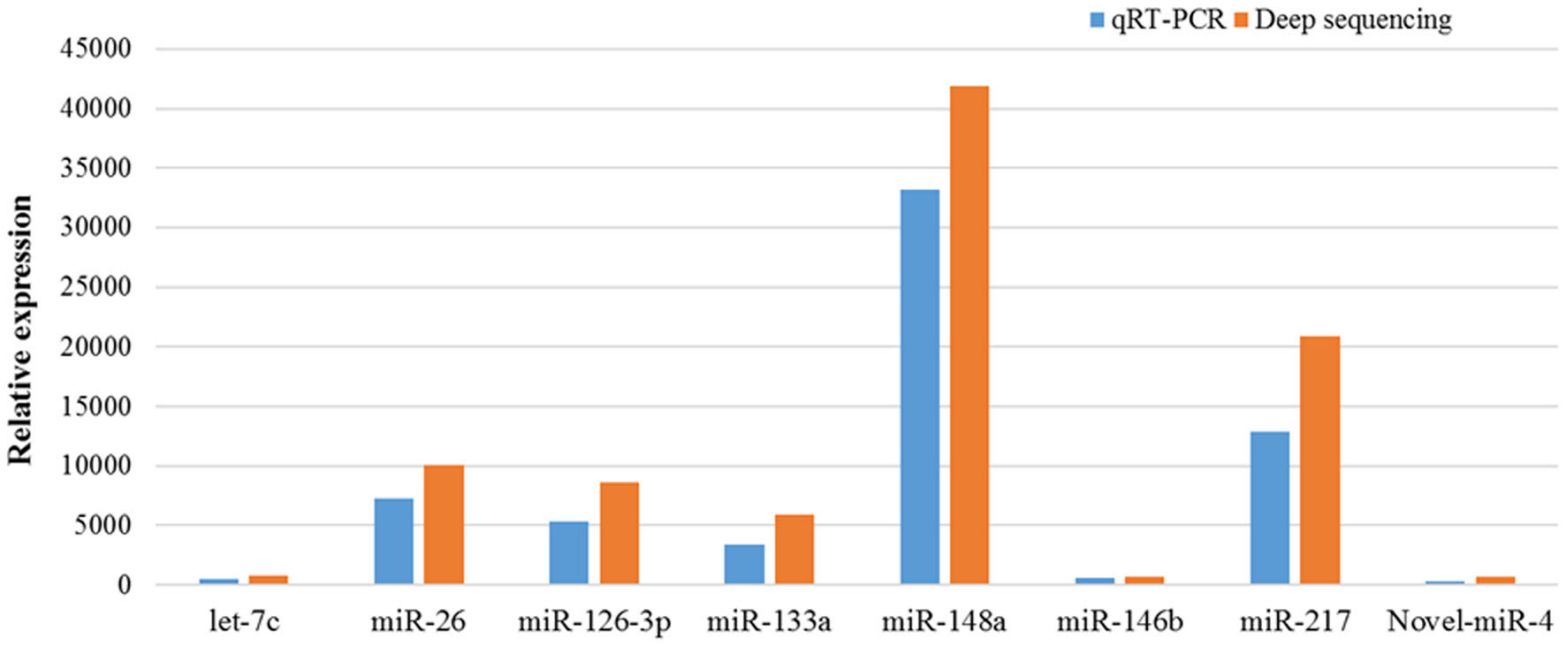
Stem-loop qPCR validation of the identified miRNAs in* A. davidianus* using deep sequencing

To validate the existence of the predicted *A. davidianus* miRNAs, the RNA samples used for the deep sequencing were subjected to stem-loop qRT-PCR. The expression levels of randomly selected miRNAs were determined, including five miRNAs (miR-26, miR-126-3p, miR-133a, miR-148a, and miR-217) with more than 5000 higher read numbers, two miRNAs with less than 600 lower read numbers (let-7c and miR-146b), and one *A. davidianus*-specific miRNAs with 334 read numbers (novel-miR-4). The qPCR results are shown in Fig. 4. The expression levels of miRNAs were represented using the normalized fold expression. The expression pattern of miRNAs was consistent with the deep sequencing results, indicating that the deep sequencing data were available and reliable.

### Prediction of the target genes for miRNAs and functional enrichment analysis

**Fig. 5:**
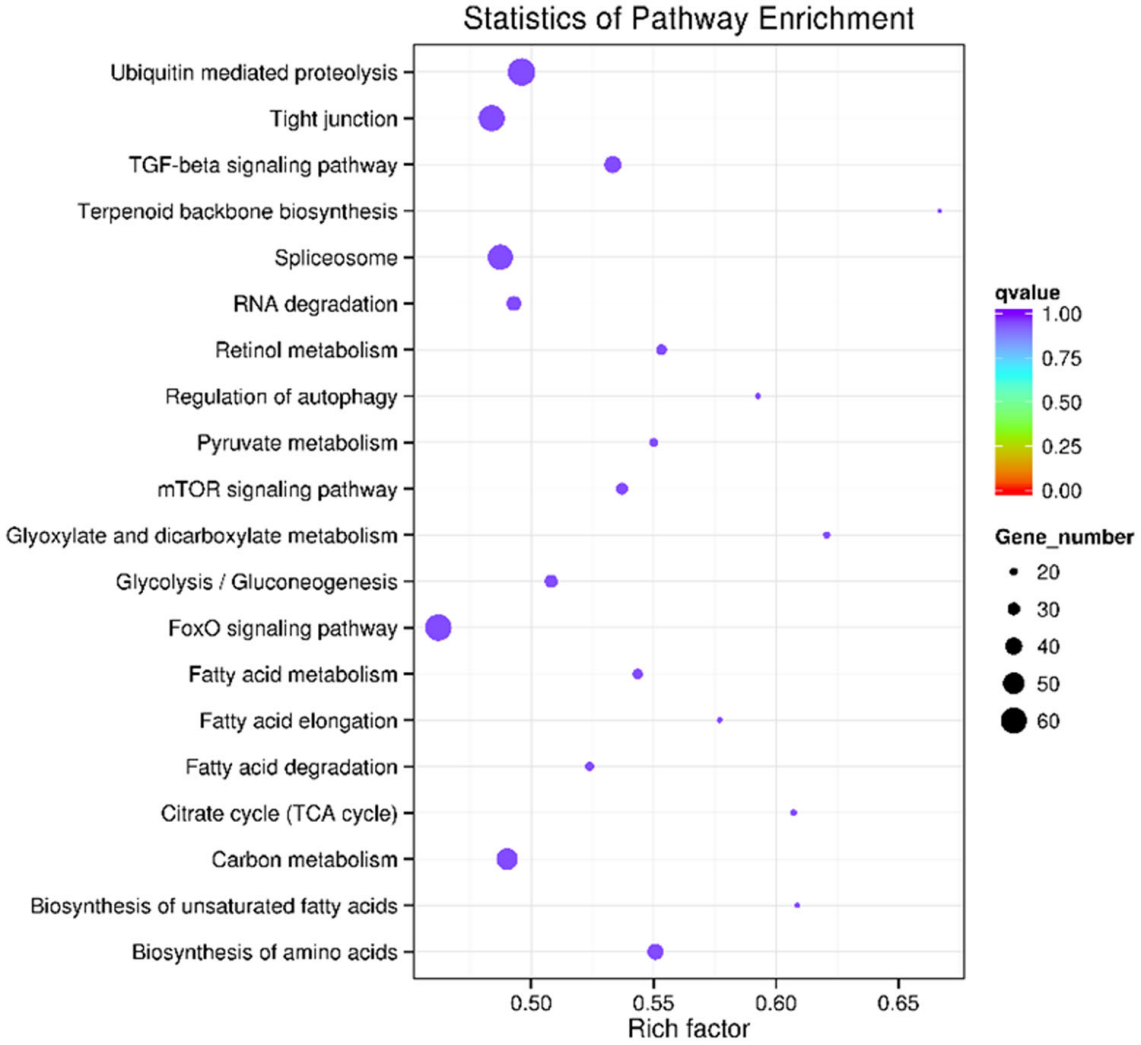
KEGG analysis with the 20 most enriched pathways. The *coloring* of the *q* values indicates the significance of the rich factor; the *circle* indicates the target genes that are involved, and the size is proportional to the gene number

The sequence complementarity between miRNAs and their corresponding target genes offer a straight forward process with which can search miRNAs target genes efficiently. A total of 4.700 annotated mRNA transcripts were found for 143 miRNAs using miRanda software (Table S8). The number of target genes for each differentially expressed miRNAs ranged from 1 to more than 100. Not surprisingly, a single miRNA can regulate multi-genes, and similarly multiple miRNAs can regulate a single gene, indicating that the miRNA gene regulation network might be extremely complicated. Most of predicted targets are transcription factors, such as those encoding the zinc finger, exonuclease domain, ATPase domain, TATA-box binding protein, EGF-like domain, PDZ domain, Rab-GTPase-TBC domain, CP2 transcription factor, and so on, which may play critical regulatory roles in growth and development of *A. davidianus*. To gain a global overview of the regulatory functions of miRNAs, the GO terms of all targets were analyzed. These targets were identified for the top 60 enriched GO categories in terms of biological process, cellular component, and molecular function (Fig. S2). Based on the biological process, the genes were classified into 20 categories of which the top 4 over-represented GO terms were developmental process, multicellular organismal development, single-organism developmental process, and anatomical structure development. In the case of cellular component, the genes were classified into 20 groups, and more than 30% of all predicated target genes were clustered into the intracellular, intracellular part, and intracellular organelle. Based on molecular function, the genes were classified into 20 categories, of which the top two were related to the nucleic acid binding and DNA binding. To universally summarize the orchestrating roles of miRNAs in *A. davidianus*, enriched KEGG analysis of target genes of identified miRNAs was performed. A total of 155 highly diversified biochemical pathways are involved with identified miRNAs target genes, considering *q* values and the number of involved genes (Table S9). Moreover, the top 20 enriched pathways were discovered with 652 involved genes (Fig. 5; Table S10). Ubiquitin-mediated proteolysis is the most significantly enriched with respect to the rich factor and gene number (63 genes), followed by FOXO signaling pathway (61 genes), tight junction (60 genes), and spliceosome (58 genes). The results also highlighted pathways relating to growth, development, and cell immune, suggesting that the genes expressed in cell proliferation, differentiation, and immunity were regulated by these miRNAs. In summary, the annotations for the putative target genes imply that different metabolic patterns are regulated by the identified miRNAs of *A. davidianus*, and we will further determine how they participate in metabolism.

## Electronic supplementary material

Below is the link to the electronic supplementary material.

Supplementary material 1 (DOCX 247 kb)

Supplementary material 2 (DOC 44 kb)

Supplementary material 3 (XLS 22 kb)

Supplementary material 4 (XLSX 9 kb)

Supplementary material 5 (XLSX 9 kb)

Supplementary material 6 (XLSX 10 kb)

Supplementary material 7 (XLSX 20 kb)

Supplementary material 8 (XLSX 65 kb)

Supplementary material 9 (XLSX 13 kb)

Supplementary material 10 (XLSX 396 kb)

Supplementary material 11 (XLSX 57 kb)
