# Supplementary material for: Identification and characterization of the Chinese giant salamander (Andrias davidianus) miRNAs by deep sequencing and predication of their targets
Source: 3 Biotech. 2017 Jul 10;7(4):235. doi: 10.1007/s13205-017-0817-3 (PMC5503845; doi:10.1007/s13205-017-0817-3)
Supplement: Supplementary file 1 — Supplementary material 1 (DOCX 247 kb) [file 13205_2017_817_MOESM1_ESM.docx]

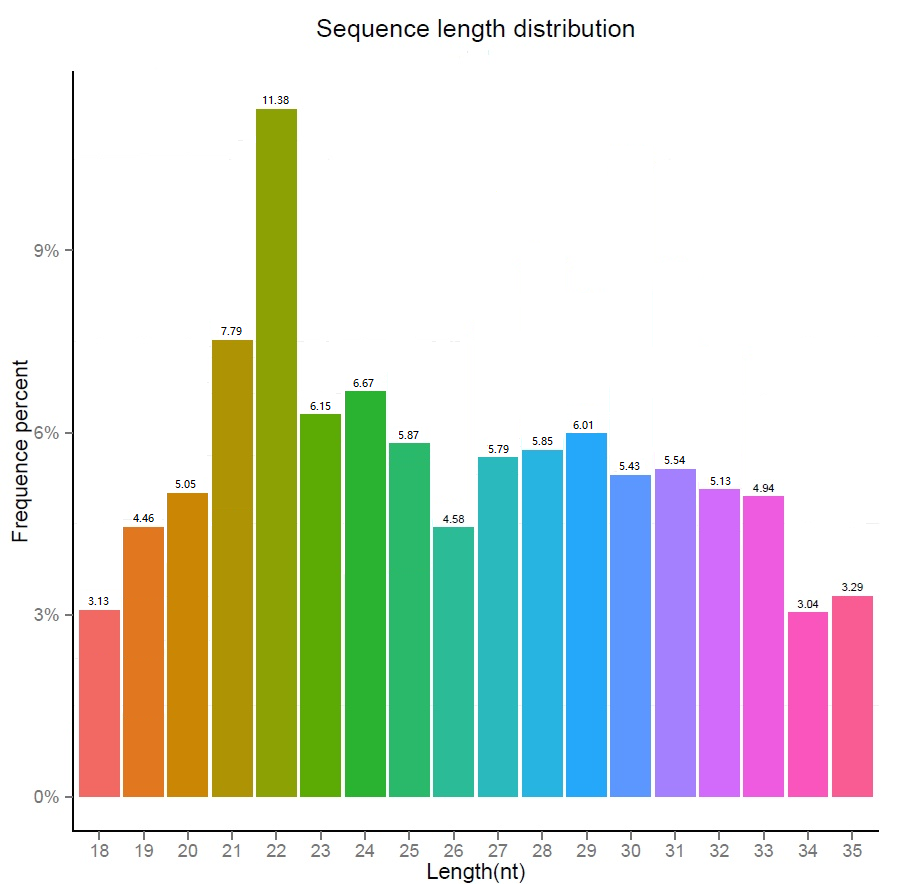


Figure S1. Length distribution of small RNAs from*A. davidianus*identifiedand analyzed by deep sequencing.


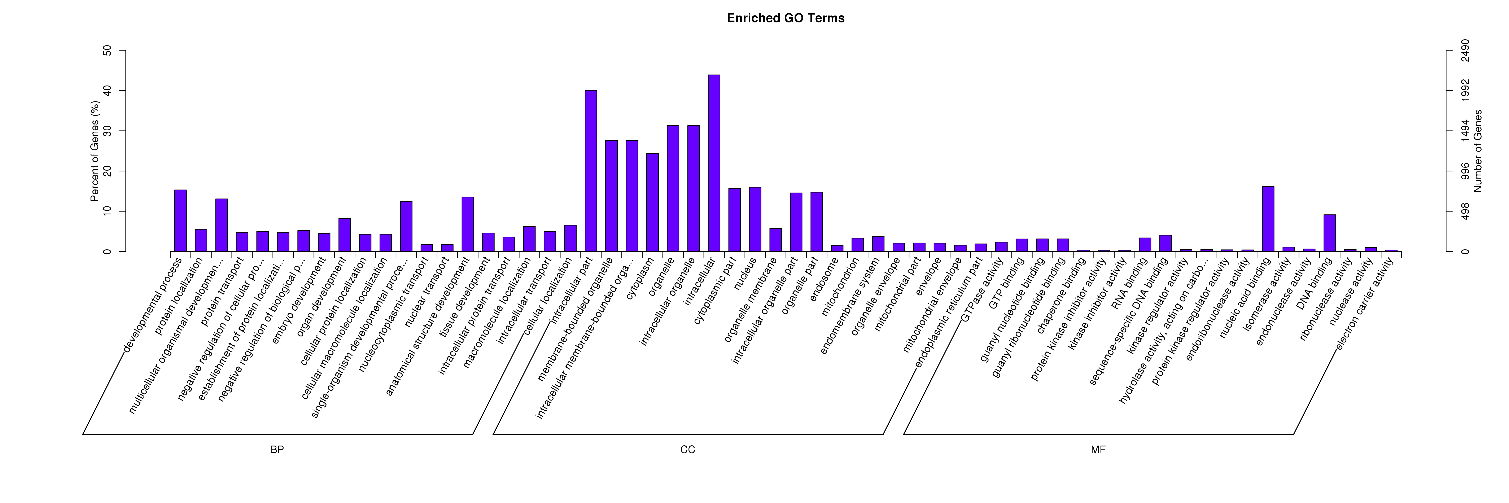


Figure S2. GO terms and numbers of the predicted target genes for identified miRNAs in *A. davidianus*. BP, Biological Process; CC, CellularComponent; MF, Molecular Function.
