# Supplementary material for: Identification and characterization of the Chinese giant salamander (Andrias davidianus) miRNAs by deep sequencing and predication of their targets
Source: 3 Biotech. 2017 Jul 10;7(4):235. doi: 10.1007/s13205-017-0817-3 (PMC5503845; doi:10.1007/s13205-017-0817-3)
Supplement: Supplementary file 2 — Supplementary material 2 (DOC 44 kb) [file 13205_2017_817_MOESM2_ESM.doc]

**Table S1. Forward, stem-loop and universal primers used to amplify miRNAs and 18srRNA in qPCR.**

| **Primer** |  | **Sequence** |
| --- | --- | --- |
| **let-7c** | **Stem**  **Fwd**  **Rev** | 5’- GTCGTATCCAGTGCAGGGTCCGAGGTATTCGCACTGGATACGACAACCAT-3’  5’- CGCATGAGGTAGTAGGTTGT-3’  5’- GTGCAGGGTCCGAGGT -3’ |
| **miR-26** | **Stem**  **Fwd**  **Rev** | 5’- GTCGTATCCAGTGCAGGGTCCGAGGTATTCGCACTGGATACGACGCCTAT-3’  5’- GCGATTCAAGTAATCCAGG -3’  5’- GTGCAGGGTCCGAGGT -3’ |
| **miR-126-3p** | **Stem**  **Fwd**  **Rev** | 5’- GTCGTATCCAGTGCAGGGTCCGAGGTATTCGCACTGGATACGACGCATTA-3’  5’ - CGCATCGTACCGTGAGTAA -3’  5’- GTGCAGGGTCCGAGGT -3’ |
| **miR-133a** | **Stem**  **Fwd**  **Rev** | 5’- GTCGTATCCAGTGCAGGGTCCGAGGTATTCGCACTGGATACGACACAGCT -3’  5’- GGCAGTTGGTCCCCTTCAACC -3’  5’- GTGCAGGGTCCGAGGT -3’ |
| **miR-148a** | **Stem**  **Fwd**  **Rev** | 5’- GTCGTATCCAGTGCAGGGTCCGAGGTATTCGCACTGGATACGACACAAAG -3’  5’- CGCGCTCAGTGCACTACAGAA -3’  5’- GTGCAGGGTCCGAGGT -3’ |
| **miR-146b** | **Stem**  **Fwd**  **Rev** | 5’- GTCGTATCCAGTGCAGGGTCCGAGGTATTCGCACTGGATACGACAGTCCA -3’  5’- GCCGTGAGAACTGAATTCCA -3’  5’- GTGCAGGGTCCGAGGT -3’ |
| **miR-217** | **Stem**  **Fwd**  **Rev** | 5’- GTCGTATCCAGTGCAGGGTCCGAGGTATTCGCACTGGATACGACATCCAA -3’  5’- GCCTACTGCATCAGGAACTGA -3’  5’- GTGCAGGGTCCGAGGT-3’ |
| **Novel-miR-4** | **Stem**  **Fwd**  **Rev** | 5’- GTCGTATCCAGTGCAGGGTCCGAGGTATTCGCACTGGATACGACTTACAT -3’  5’- GCGCCTGGAATGTTAAGAAGT-3’  5’- GTGCAGGGTCCGAGGT-3’ |
| **18srRNA** | Fwd  Rev | 5’- CCTGAGAAACGGCTACCACATCC -3’  5’- AGCAACTTTAGTATACGCTATTGGAG-3’ |
